# Supplementary material for: Effect of an Electronic Medical Record-Based Screening System on a Rapid Response System: 8-Years’ Experience of a Single Center Cohort
Source: J Clin Med. 2020 Feb 1;9(2):383. doi: 10.3390/jcm9020383 (PMC7073515; doi:10.3390/jcm9020383)
Supplement: Supplementary file 1 [file jcm-09-00383-s001.pdf]

Table S1. Criteria for medical alert team activation

**Screening criteria from electronic medical record**	
Systemic mean blood pressure < 60 mm Hg or systolic blood pressure < 90 mm Hg	
Respiratory distress (rate > 25 or < 8 breaths/min)	
Unexplained pulse rate > 130 beats/min or pulse rate < 50 beats/min	
Unexplained metabolic acidosis (pH < 7.3) or lactate > 2 mmol/L	
$PaCO_2$ > 50 mmHg or PaO_2 < 55mmHg	
Glucose < 2.8 mmol/L	
Sudden mental change or unexplained agitation	
Applying O_2 nasal prong > 3 L, or venturi mask > 30%	
Unexplained seizures	
Chest pain	
Upper airway obstruction sign: stridor	
**Calling criteria**	
Airway	Threatened or stridor
Breathing	Respiratory rate < 6 breaths/min or Respiratory rate > 30 breaths/m
	SpO_2 < 90% on venturi mask 40% or O_2 12 L/min
Circulation	Pulse rate < 40 beats/min Pulse rate > 140 beats/min
	Systolic blood pressure < 90 mm Hg
Neurology	Sudden mental change or Seizure
Others	Bedside nurse's concern about overall deterioration

Table S2. Modified early weaning score (MEWS)

	**3**	**2**	**1**	**0**	**1**	**2**	**3**
**Systolic Blood pressure (mmHg)**	≤ 70	71-80	81-100	101-199		≥200	
**Pulse rate (bpm)**		≤40	41-50	51-100	101-110	111-129	≥130
**Respiratory rate (bpm)**		**< 9**		9-14	15-20	21-29	≥30
**Temperature (℃)**		<35		35-38.4		≥38.5	
**Mental status**				Alert	Reacts to voice	Reacts to pain	Unresponsiveness

Table S3. Baseline patients and event characteristics

	Daytime		On-call		Total
	**Call**	**Screening**	**Call**	**Screening**	
	N=1897	N=1836	N=2874	N=3419	N=10026
**Activation coding**					
**Sepsis**	103(5.4)	143(7.8) †	157(5.5)	331(9.7) ‡	734(7.3)
**Septic shock**	179(9.4)	235(12.8) ‡	293(10.2)	417(12.2)*	1124(11.2)
**Respiratory distress**	980(51.7)	930(50.7)	1444(50.2)	1739(50.9)	5093(50.8)
**Cardiac arrest**	33(1.7)‡	5(0.3)	45(1.6) ‡	2(0.06)	85(0.8)
**Cardiogenic shock**	31(1.6)‡	10(0.5)	32(1.1)	33(1.0)	106(1.1)
**Anaphylactic shock**	20(1.1)†	5(0.3)	15(0.5)	7(0.2)	47(0.5)
**Hypovolemic shock**	143(7.5) ‡	64(3.5)	277(9.6)‡	169(4.9)	653(6.5)
**Obstructive shock (PTE)**	5 (0.3)	2(0.1)	7(0.2)	7(0.2)	21(0.2)
**Arrhythmia**	24(1.3)	22(1.2)	65(2.3)	56(1.6)	167(1.7)
**Altered mentality**	207(10.9)‡	44(2.4)	236(8.2)‡	84(2.5)	571(5.7)
**Metabolic acidosis**	50(2.6)	248(13.5)‡	68(2.4)	290(8.5)‡	656(6.5)
**(old) Sepsis**	24(1.3)	30(1.6)	43(1.5)	76(2.2)	173(1.7)
**Hypertension**	42(2.2)	62(3.4)*	79(2.7)	136(4.0)†	319(3.2)
**Others**	56(3.0)	36(2.0)	113(3.9)‡	72(2.1)	277(2.8)

Categorical variables are presented as No. (%).*p - value < 0.05, †p - value < 0.01, ‡p - value < 0.001. Chi-square test was done for the comparison between daytime calling and daytime screening. Same analytic technique was used for the comparison between on-call calling and on-call screening. PTE = pulmonary thromboembolism.

Table S3. Baseline patients and event characteristics. Continued

	Day time		On-call		Total
	Call	Screening	Call	Screening	
	N=1897	N=1836	N=2874	N=3419	N=10026
**Intervention**					
**ACLS**	44(2.3)‡	10(0.5)	77(2.7)‡	6(0.2)	137(1.4)
**ECMO**	2(0.1)	1(0.0)	1(0.0)	0(0.0)	4 (0.04)
**Intubation**	424(22.4)‡	115(6.3)	575(20.0)‡	183(5.4)	1297(12.9)
**Difficult airway**	22(1.2)†	6(0.3)	29(1.0)†	12(0.4)	69(0.7)
**Ventilator**	205(10.8)‡	51(3.4)	217(7.6)‡	78(2.3)	562(5.6)
**BiPAP**	65(3.4)†	64(1.9)	91(3.2)‡	44(1.3)	234(2.3)
**HFNC**	96(5.1)	107(5.8)	149(5.2)	251(7.3)‡	603(6.0)
**O2**	503(27.8)‡	291(16.1)	721(25.9)‡	574(17.2)	2089(21.5)
**BFS**	8(0.4)*	1(0.06)	22(0.8)‡	2(0.06)	33(0.3)
**Fiberoptic BFS**	6(0.3)	4(0.2)	16(0.6)‡	2(0.06)	28(0.3)
**T-can**	14(0.8)*	5(0.3)	19(0.7)*	9(0.3)	47(0.5)
**Suction**	401(22.2)‡	146(8.1)	568(20.4)‡	271(8.1)	1386(14.2)
**Sepsis shock**	235(13.0)	308(17.1)‡	363(13.1)	604(18.1)‡	1510(15.5)

Categorical variables are presented as No. (%).* p - value < 0.05, †p - value < 0.01, ‡p - value < 0.001. Chi-square test was done for the comparison between daytime calling and daytime screening. Same analytic technique was used for the comparison between on-call calling and on-call screening. ACLS = advanced cardiovascular life support; BiPAP = bilevel positive airway pressure; BFS = bronchoscopy; ECMO = extracorporeal membrane oxygenation; HFNC = high flow nasal cannula; T-can = tracheal cannula.

Table S4. The multivariate analysis for the risk factors associated with ICU admission and 28-day mortality after RRT activation

Variables	OR	95% CI	p-value	Variables	OR	95% CI	p-value
**ICU admission**							
**Illness type**				**Activation coding**			
Medical	1			Sepsis	1	1	
Surgical	1.78	1.54-2.05	<0.001	Septic shock	2.82	2.18-3.66	<0.001
				Respiratory distress	1.4	1.11-1.77	0.005
**MEWS**	1.09	1.06-1.12	<0.001	Cardiogenic shock	3.78	2.33-6.11	<0.001
				Hypovolemic shock	1.53	1.14-2.06	0.004
**Time**				Obstructive shock (PTE)	3.74	1.35-10.34	0.011
Daytime	1			Altered mentality	1.51	1.11-2.05	0.008
On-call	0.76	0.68-0.84	<0.001	Metabolic acidosis	1.36	1.02-1.92	0.039
				Hypertension	0.41	0.24-0.68	<0.001
**Trigger type**							
Calling	1						
Screening	0.51	0.46-0.57	<0.001				
**28-day mortality**							
**Illness type**				**Activation coding**			
Medical	1			Sepsis	1		
Surgical	0.32	0.74-0.38	<0.001	Septic shock	1.57	1.24-1.99	<0.001
				Respiratory distress	2.27	1.85-2.80	<0.001
**MEWS**	1.15	1.12-1.17	<0.001	Cardiac arrest	2.89	1.25-6.68	0.013
				Cardiogenic shock	1.77	1.08-2.91	<0.001
**Time**				Anaphylactic shock	0.2	0.05-0.83	0.027
Daytime	1			Hypovolemic shock	1.81	1.38-2.39	<0.001
On-call	0.85	0.78-0.94	0.001	Altered mentality	2.24	1.70-2.94	<0.001
				Metabolic acidosis	3.00	2.32-3.90	<0.001
**Trigger type**				Hypertension	0.60	0.38-0.82	0.021
Calling	1						
Screening	1.19	1.08-1.31	0.001	**Intervention**			
				ACLS	7.03	3.94-12.55	<0.001
**Underlying disease**				Ventilator	1.44	1.18-1.75	<0.001
Solid malignancy	2.67	2.40-2.97	0.001	HFNC	1.66	1.37-1.99	<0.001
Hematologic malignancy	2.11	1.84-2.42	0.001	O2	1.21	1.08-1.36	0.001
Chronic liver disease	1.79	1.58-2.04	0.001				
Chronic kidney disease	1.26	1.03-1.54	0.023				

Data are presented as odds ratio (OR) with 95% confidence interval (CI). See Table 1 and Table S3 legend for expansion of abbreviations. For ICU admission, illness type, MEWS, Time, Trigger type, and activation code were selected for variables for multivariate logistic regression model. For 28-day mortality, illness type, MEWS, Time, Trigger type, underlying disease, activation code, and intervention were variables selected for multivariate logistic regression model.
